# Supplementary material for: A systematic review and meta-analysis of human and zoonotic dog soil-transmitted helminth infections in Australian Indigenous communities
Source: PLoS Negl Trop Dis. 2022 Oct 24;16(10):e0010895. doi: 10.1371/journal.pntd.0010895 (PMC9632820; doi:10.1371/journal.pntd.0010895)
Supplement: Attachment — Submitted filename: Response letter.docx [file pntd.0010895.s007.docx]

Editors

PLOS Neglected Tropical Diseases

31/05/2022

Cameron Raw
Faculty of Veterinary and Agricultural Science

University of Melbourne

Parkville, VIC 3052

Australia

Dear Drs Mahanty and Karunaweera,

Please find a revised version of our manuscript “A systematic review and meta-analysis of human and zoonotic dog soil-transmitted helminth infections in Australian Indigenous communities” for reconsideration by PLOS Neglected Tropical Diseases. Our responses to the reviewers’ comments are detailed below.

The authors would like to thank the reviewers for their constructive reviews, and hope that the manuscript is now at a standard suitable for publication in PLOS Neglected Tropical Diseases as a research article.

Sincerely,

Cameron Raw, BVSc (Hons), PhD Candidate

**Methods**

**Reviewer 2 comments:**

*Table 1 Legend: table legends should stand alone, please provide more detail in the figure legend of what this table shows.*

Table 1 legend now modified to read “Table 1: Systematic review search terms ^a^” with a legend reading “search terms combined sing Boolean logic, with row terms combined using ‘or’ statements and ‘and’ statements used to combine terms between rows”

*Also, with reference to Table 2:*

*a) In my experience, the sensitivity of flotation techniques for Strongyloides is much lower. In fact, the reference given for the diagnostic sensitivity of zinc-sulfate flotation reports combined data of both zinc sulfate flotation and formalin -ethyl acetate flotation (one or the other). This is not a suitable reference to use for these calculations. Please find an alternative, suitable, reference and re-calculate your true prevalence data for this methodology.*

We agree. There is scant evidence of the sensitivity and specificity of SSF for the detection of *Strongyloides*, likely due to its poor performance as a diagnostic test for this parasite. However, a study by Schär *et al.* (2014) reveals its accuracy in relation to the Baermann method, with a calculable sensitivity of 9.3%. This figure has now been included in the table along with the reference, and new TP estimates have been calculated, corrected in the manuscript as well as in Figure 4.

*b) Can you state that this is data for the commonly used Verweij PCR? There are several, each with unique sensitivity and specificity, was this the only method used in all the papers reviewed?*

We can confirm that all conventional PCR methods for hookworm used in papers included in meta-analysis referred to the method published in the following paper: Traub RJ, Inpankaew T, Sutthikornchai C, Sukthana Y, Thompson RCA. PCR-based coprodiagnostic tools reveal dogs as reservoirs of zoonotic ancylostomiasis caused by Ancylostoma ceylanicum in temple communities in Bangkok. *Vet Parasitol*. 2008. Only one paper eligible for inclusion featured qPCR for the detection of *Strongyloides* *stercoralis* (Beknazarova *et al.* 2019), which used the method from Verweij *et al.* (2009). These are detailed in the ‘Diagnostic/identification details’ column of the S1 Table.

*c) Which ELISA serology? As above, sensitivity and specificity of ELISA kits vary by serological antigen (and has not been reported for some in-house developed kits). If within an acceptable range, you may be able to use the one value to determine TP, but this should be considered and addressed in the review.*

The study by Miller, Young and Tye (2018) did not specify the serological method used, however, did provide reference to an IFAT. The 2014 study by Einsiedel *et al.* used an IgG ELISA based on *Strongyloides* *ratti*, as did the studies by Page *et al.* (2006), Hays *et al.* (2015), Shield *et al.* (2015) and Kearns *et al.* (2017). Four other studies by Einsiedel *et al.* were included in meta-analysis but did not specifically state the serological method used. However, given these studies were written by the same primary author, were from the same hospital study location and were from adjacent study timeframes, it is highly likely that the same *S. ratti* IgG ELISA was used.

While the authors acknowledge that cut-offs, study populations and methods can have significant impacts on serological sensitivity and specificity, published data such as that of Bisoffi *et al.* (2014) and Requena-Méndez *et al.* (2013) indicate that the sensitivity and specificity of the tests included in this meta-analysis do not differ significantly.

This is addressed in the revised manuscript, lines 235-244:

“*S.* *stercoralis* serologic tests can vary widely in sensitivity and specificity based on cut-offs, study populations, reference methods and test methods [48]. Five of the ten studies included for meta-analysis stated the use of IgG ELISA. A further four studies published by common authors did not refer to serological methods used but utilised the same study population and adjacent study timeframes, and thus are assumed to have used the same IgG ELISA assay. Another study did not specifically state the serological method used to diagnose *S.* *stercoralis* exposure but referenced a study in which indirect immunofluorescence test (IFAT) was utilised. Given that these methods have very similar reported sensitivities and specificities in the source studies referenced in Table 2, the average diagnostic parameters were used in calculations of TP.”

**Results**

**Reviewer 2 comments:**

*Line 245: grammar – “fitted”*

Changed to ‘fitted’

*Lines 267-268: Please provide the reference for this study from the Kimberley which identified A. lumbricoides infections. Perhaps also note that the Kimberley is in North-West Western Australia (for international readers who are not immediately aware of various regions of Australia)?*

Changed to ‘*Ascaris lumbricoides* has been omitted from mapping as only one case was reported in a 1992 study from the Kimberley region in North-West Western Australia [reference to Gunzberg *et al.* (1992).]’

*Line 285: What about true dingos (not hybridised)? Might any of these have been included in the study?*

Changed for clarity to read ‘Wild dogs and dingoes may be dogs living wild existences, dog/dingo hybrids or pure dingoes, though distinctions were not made in the included studies.’

*Line 291-292: Can you break down which serological kits were used (in-house, commercial, antigen type) and put this in a supplementary table? This has relevance to the prevalences reported – this data may be included as a column in the S1 Table.*

Test details have been added to the ‘Diagnostic/identification details’ column in the revised S1 Table file as per the serological study details above

*Lines 293, 296: Ditto for conventional PCR and qPCR methodologies.*

These have also been added under the ‘Diagnostic/identification details’ in the revised S1 Table file

*Table S1 needs a superior legend – what do the green and amber colours in some of the columns mean? What does L, M and H mean (low, medium, and high? Referring to what? For instance, columns are titled things like, “Random or census” and “length of shortest prevalence period appropriate?”. Results are then noted as “low”, “medium” or “high”– shouldn’t this be listed “random” or “census”; or as a length of shortest prevalence period (depending on the column)?? I cannot determine how to interpret some aspects of this table.*

These items refer to the risk of bias tool published by Hoy *et al.* as noted on line 192 of the ‘Data extraction’ section of the review. A prefix of ‘Risk of bias’ has been attached to all columns, and row entries have been modified from ‘L’ to ‘Low risk’ and from ‘H’ to ‘High risk’. Additionally, for clarity, a new tab titled ‘Metadata’ has been created in the revised S1 Table file which provides more detail on all columns in the table, refers to the Hoy *et al.* risk of bias tool in greater detail, and specifies what constitutes high or low risk in the included studies as per the Hoy *et al.* tool methodology.

*Figure 3: Please include the sampling dates that these data span for each species*

Title of each map has been amended to include sampling date spans for each species, and Figure 3 caption has been revised to ‘Fig 3. Detected STH species and sampling date spans in domestic dog, wild dog, dingo and human hosts in relation to Australian Indigenous community locations.’

**Conclusions**

**Reviewer 2 comments:**

*Lines 402-412: Just a comment for the author's information and consideration here, which the authors may wish to include in this discussion paragraph or not include (it is not essential to the text). There was once, and still is in some circles, an incorrect belief that all human hookworms in Australia were due to A. duodenale. So, all hookworm infections in humans have been assigned to this species in many papers, particularly circa 1970-2000. Prociv found Necator in Australia and two human A. ceylanicum infections have been identified, so clearly this assumption was incorrect.*

We agree that hookworm eggs assumed to be *A. caninum* in dogs and *A. duodenale* in humans may have been misclassified in Australia as eggs are morphologically identical for all species of *Ancylostoma*. To make this clear, we have amended lines 440-443 to read:

‘In the case of hookworm species *A. caninum* in dogs and *A. duodenale* in humans, coproscopic methods examining eggs including SSF, FE and DS are unable to determine species due to the identical morphological appearance of *Ancylostoma* spp. eggs.’

*Line 444: Can a more scientific term than “ill thrift” be used?*

Modified to read ‘Missed infections with hookworm or whipworm may lead to protracted anaemia, malaise and stunting in younger humans and animals, or in the case of *S. stercoralis*, hyperinfection can lead to severe disease and death.’

**Editorial and Data Presentation Modifications**

**Reviewer 2 comments:**

Abstract & Author Summary:

*Lines 20-27: please make clear if these pooled prevalence rates are for dogs or humans. I assume dogs as you note A. caninum, but on initial reading, this is not clear, as later you mention T. trichiura – please differentiate the relevant host species for these results.*

As this review is considering these parasite species from a zoonotic and One Health perspective, these results are presented from the pooled true prevalence estimates of all host species for each parasite. However, the combination of host species results to establish pooled prevalence only occurs in the case of *S.* *stercoralis*, as seen in Figure 4 (combining 30 human prevalence studies and a single prevalence study in dogs). The abstract has been modified to include the contributing host species after each parasite species mentioned.

*Line 36: can you rewrite to make clear that “zoonotic” means they cross between humans and animals?*

Line modified to read: ‘These worms may infect different hosts including humans and dogs, and some species are zoonotic meaning that they able to cross between animals and humans.’

Introduction:
*Line 49 – perhaps “are Neglected Tropical Diseases” instead of “belong to”?*

Line modified to read: ‘Soil-transmitted helminths (STHs) are Neglected Tropical Diseases (NTDs) and infect an estimated 1.5 billion people and countless animals worldwide.’

*Line 52: “may act as zoonotic reservoirs” – it depends on the STH if they do*

Line modified to read: ‘…other hosts such as dogs, that may act as zoonotic reservoirs for STHs…’

*Line 57: please add A. ceylanicum to the list of hookworms*

Line modified to read: ‘STHs relevant to humans in Australia include hookworms (including *Ancylostoma duodenale, Ancylostoma ceylanicum* and *Necator americanus*)…’

*Line 65-67: I think this sentence needs to be rewritten somewhat as it reads as though S. stercoralis is a confirmed zoonosis from cats and dogs. This is probably, but not yet proven.*

This line has been modified so that the mention of *S.* *stercoralis* has been removed here. This is addressed later in the paragraph with the sentence ‘Strongyloidiasis is also a potential zoonosis, with S. stercoralis comprising two distinct genetic clades, one restricted to dogs and another infecting humans, non-human primates, dogs and cats’. This more accurately reflects current evidence.

*Line 84-85: It is not the motility of L3 hookworm and Strongyloides that allows them to infect their hosts percutaneously, but rather the enzymes they produce which allow skin penetration. This sentence should be reworded to avoid the misconception that motility alone is responsible for percutaneous penetration.*

Line modified to read simply: ‘Third stage filariform larvae of all hookworm species and *Strongyloides* also infect their host by percutaneous penetration of exposed skin in contact with contaminated matter’

*Line 107: “…in Australian Aboriginal communities” – please provide the context of where this One Health understanding is required due to culturally relevant bonds.*

Line modified to read: ‘These risks and the importance of the human-animal bond in Australian Indigenous communities necessitates a culturally relevant One Health understanding of these infections’

References:
*Some references are incomplete or not in PLoS NTDs format (e.g. Lima & Delgado). Please review and correct the reference title list as necessary.*

These have been reviewed and corrected as requested

**Summary and General Comments**

**Reviewer 1 comments:**

*Line 11: I wonder how good have the control efforts been*

We agree and also wonder how effective these have been. Surveillance certainly seems to have been greater during the Australian Hookworm Campaign, but efforts of eradication seem to have been mostly focussed on non-Indigenous populations at that time. More recent efforts appear to have been more sporadic, with limited funding going to other – possibly more pressing – priorities.

Introduction
*Line 59: Ascaris is rare in Australia – has it been found in remote communities in the last few decades - so not sure if it is super relevant.*

We agree that *Ascaris* infection is rare, however in this case the title sentence of the paragraph refers to those parasites which have been detected as infecting humans in Australia.

*Line 72: italics S. stercoralis*

Italicised as suggested

*Line 95-97: reference for this sentence*

Reference added

*Line 109: Spp. for Strongyloides as well. comma after intestines and colon.*

Modified as suggested

*Was the meta-analysis performed on a specific question? Which was? Prevalence in humans and dogs in Australia? Should state along with the systematic review goals.*

The specific aims of the review and meta-analysis are detailed in the final paragraph of the introduction (line 163 of the modified manuscript), which includes: ‘…provide an estimate of the individual study and pooled true prevalence of STH infections in Australian Indigenous communities’

*I think the introduction needs to be tightened up overall. Maybe just re-organised. Perhaps add subheadings and organise that way. I’m not sure all the information is totally necessary, and perhaps some of this information could be moved to discussion. So, a shorter introduction, go straight into methods and results, then use some of this info to put in to context.*

Introduction reorganised under ‘parasite biology’, ‘environmental factors’ and ‘socio-economic factors’ subheadings.

Methods
*211: authors or independent?*

One author and one independent veterinary parasitologist.

*Table 2: This is obviously restricted to what methods were performed in the included papers, but really no agar plate or Baermann methods were used in the included papers? Thinking specifically of strongyloides.
Strongyloides stool ID is based primarily on larvae as eggs tend to hatch in the gut – very heavy infections you may still get eggs in the stool, but lyse very quickly (as do the hookworm eggs), when DS, FE, and SSF is being performed in these studies are they doing morphological ID of the larvae, and at what stage of larvae? FE and SSF are fixatives, and presumably DS is being done immediately, obviously they are not going to be looking totally at L3 larvae.*

Larval culture was used in a small subset of samples to confirm *S.* *stercoralis* in three included studies as detailed in lines 464-468 of the manuscript. These samples had already tested positive by other means and thus inclusion would not contribute to individual or pooled true prevalence estimates in an unbiased way. The Baermann method was not used in any studies eligible for inclusion. Details of parasite stages detected from included studies are listed in the ‘Diagnostic/identification details’ column of the revised S1 Table.

*Line 299: what year was the Ascaris study published?*

This study was published in 1992. This and further data on this study by Gunzburg *et al.* is available in the S1 Table file. Refence to the date of this study is also now made in the results section on line 303.

*Line 426-428: Hmmm makes you really doubt if it is strongyloides in the one’s that don’t use these techniques to confirm (if they used one of the other microscopic techniques initially and not ELISA or PCR)*

We agree, this calls into question whether *Strongyloides* was truly detected. The issues with Strongyloides detection are discussed in the discussion section of this manuscript (revised manuscript line 456 onwards)

*Line 438: was it mentioned in methods that analysis was done in two time periods for this figure?*

This does not refer to the methodology of the meta-analysis, but rather the fact that studies eligible for inclusion in meta-analysis were published between 1993-2018 in the case of *A. caninum* and 1976-2019 in the case of *S.* *stercoralis*. These dates are included in Figure 4. This line has been modified to ensure that this is clearer.
