# Supplementary material for: A systematic review and meta-analysis of human and zoonotic dog soil-transmitted helminth infections in Australian Indigenous communities
Source: PLoS Negl Trop Dis. 2022 Oct 24;16(10):e0010895. doi: 10.1371/journal.pntd.0010895 (PMC9632820; doi:10.1371/journal.pntd.0010895)
Supplement: Attachment — Submitted filename: Response letter.docx [file pntd.0010895.s008.docx]

Editors

PLOS Neglected Tropical Diseases

31/05/2022

Cameron Raw
Faculty of Veterinary and Agricultural Science

University of Melbourne

Parkville, VIC 3052

Australia

Dear Drs Mahanty and Karunaweera,

Please find a revised version of our manuscript “A systematic review and meta-analysis of human and zoonotic dog soil-transmitted helminth infections in Australian Indigenous communities” for reconsideration by PLOS Neglected Tropical Diseases. Our responses to the reviewers’ comments are detailed below.

The authors would like to thank the reviewers for their constructive reviews, and hope that the manuscript is now at a standard suitable for publication in PLOS Neglected Tropical Diseases as a research article.

Sincerely,

Cameron Raw, BVSc (Hons), PhD Candidate

**Editorial and Data Presentation Modifications**

**Reviewer 2 comments:**

*Line 110: While S. stercoralis infections are self-limiting clinically for dogs, I have not seen evidence for this in humans, please check this statement is correct for human hosts and amend if necessary*

Amended to ‘This is important because while most *S. stercoralis* cases are self-limiting in dogs the non-specific clinical presentation that may manifest in both dogs and humans, can potentially progress rapidly to a state of hyperinfection or disseminated strongyloidiasis, which constitute a poor prognosis.’

*I cannot see the changes in table S1 that are described in the response to reviewers. Perhaps an old version of Table S1 has been uploaded?*

The amended table has been uploaded in the previous submission with the filename ‘S1_Table_revised.xlsx’ and contains all of the changes noted in the previous submission. These changes were: greater detail in risk of bias columns (columns V-AI); a metadata tab detailing all column headings and entries with extra details on how risk of bias was determined; and greater diagnostic test details and sources for methodologies (column O).

**Summary and General Comments**

**Reviewer 1 comments:**

*Line 69: space caninum[6-9]*

Amended as suggested.

*Line 69: A. caninum*

Amended as suggested.

*Line 87-88: Consider moving this to after the next line about third stage larvae penetrating. As my first thought on reading ‘in the same manner’ was, well mostly they penetrate the skin directly.*

Amended as suggested to put this sentence later, along with other small edits for clarity. This section now reads:

‘Third stage filariform larvae of all hookworm species and *Strongyloides* can infect their host by percutaneous penetration of exposed skin in contact with contaminated matter. People who regularly walk barefoot outdoors, as regularly observed in remote Australian Indigenous communities, demonstrate higher risk of STH infection. A Malaysian study found that humans who routinely walked barefoot outdoors were 5.6 (95% CI 2.9 – 10.7) times more likely to be infected with hookworms compared with those who routinely wore shoes. *T. trichiura* and *A. lumbricoides* infect their human definitive host by ingestion of substrates contaminated with embryonated eggs. Third-stage filariform larvae of some *Ancylostoma* spp. can also infect their hosts via ingestion of contaminated matter. Dogs may also become infected with *A. caninum* by the ingestion of paratenic hosts such as rats or mice.’

*Line 92: all STH or just hookworm? As Ascaris is rare the STH in Australia would have to be made up of hookworm and Trichuris infection, unless the referenced article also included Strongyloides in the STH grouping which doesn’t always happen.*

Amended to read ‘People who regularly walk barefoot outdoors, as regularly observed in remote Australian Indigenous communities, demonstrate higher risk of hookworm and *Strongyloides* infection.’
